# Transcriptomic dynamics in soybean near-isogenic lines differing in alleles for an aphid resistance gene, following infestation by soybean aphid biotype 2

**DOI:** 10.1186/s12864-017-3829-9

**Published:** 2017-06-23

**Authors:** Sungwoo Lee, Bryan J. Cassone, Asela Wijeratne, Tae-Hwan Jun, Andrew P. Michel, M.A. Rouf Mian

**Affiliations:** 1 Department of Entomology, Ohio Agricultural Research and Development Center (OARDC), The Ohio State University, Wooster, OH 44691 USA; 2 Present Address: Department of Crop Science, Chungnam National University, Daejeon, 34341 South Korea; 3 Department of Plant Pathology, Ohio Agricultural Research and Development Center (OARDC), The Ohio State University, 1680 Madison Avenue, Wooster, OH 44691 USA; 4 Present Address: Department of Biology, Brandon University, Brandon, MB R7A 6A9 Canada; 5 Molecular and Cellular Imaging Center, The Ohio State University/OARDC, Wooster, OH 44691 USA; 6 Present Address: Department of Biological Sciences, University of Memphis, 3774 Walker Avenue, Memphis, TN 38152 USA; 7 Present Address: Department of Plant Bioscience, Pusan National University, Busan, 609-735 South Korea; 8 Corn, Soybean, Soft Wheat Quality Unit, USDA-ARS, Wooster, OH 44691 USA; 9 Present Address: Soybean Nitrogen Fixation Unit, USDA-ARS, 3127 Ligon Street, Raleigh, NC 27606 USA

**Keywords:** *Rag*5, RNA sequencing, *Aphis glycines* Matsumura, Aphid resistance, Near-isogenic line (NIL), DESeq2, Transcriptomic profiling, Differential gene expression

## Abstract

**Background:**

Genetic resistance of soybean [*Glycine max* (L.) Merr] against *Aphis glycines* provides effective management of this invasive pest, though the underlying molecular mechanisms are largely unknown. This study aimed to investigate genome-wide changes in gene expressions of soybean near-isogenic lines (NILs) either with the *Rag*5 allele for resistance or the *rag*5 allele for susceptibility to the aphid following infestation with soybean aphid biotype 2.

**Results:**

The resistant (R)-NIL responded more rapidly to aphid infestation than the susceptible (S)-NIL, with differential expressions of 2496 genes during first 12 h of infestation (hai), compared to the aphid-free control. Although the majority of the differentially expressed genes (DEGs) in the R-NIL also responded to aphid infestation in S-NIL, overall the response time was longer and/or the magnitude of change was smaller in the S-NIL. In addition, 915 DEGs in R-NIL continued to be regulated at all time points (0, 6, 12, and 48 hai), while only 20 DEGs did so in S-NIL. Enriched gene ontology of the 2496 DEGs involved in plant defense responses including primary metabolite catalysis, oxidative stress reduction, and phytohormone-related signaling. By comparing R- vs. S-NIL, a total of 556 DEGs were identified. Of the 13 genes annotated in a 120-kb window of the *Rag*5 locus, two genes (Glyma.13 g190200 and Glyma.13 g190600) were differentially expressed (upregulated in S- or R-NIL), and another gene (Glyma.13 g190500) was induced up to 4-fold in the R-NIL at 6 and 12 h following aphid infestation.

**Conclusions:**

This study strengthens our understanding of the defense dynamics in compatible and incompatible interactions of soybean and soybean aphid biotype 2. Several DEGs (e.g., Glyma.13 g190200, Glyma.13 g190500, and Glyma.13 g190600) near the *Rag*5 locus are strong candidate genes for further investigations.

**Electronic supplementary material:**

The online version of this article (doi:10.1186/s12864-017-3829-9) contains supplementary material, which is available to authorized users.

## Background

Soybean aphid (*Aphis glycines* Matsumura) is a phloem-feeding insect of soybean [*Glycine max* (L.) Merr] that is native to East Asia [1]. It was first found in soybean fields in northern United States in 2000 [2], and the aphid rapidly spread over the main soybean growing areas in both the United States and Canada [3]. It is a serious invasive pest of soybean in North America as well as a vector of several viruses [4]. In temperate regions, soybean aphids are heteroecious and holocyclic, where buckthorn (*Rhamnus* spp.) is the primary host plant and soybean is a secondary host [5]. Soybean aphids sexually reproduce on buckthorn, survive as eggs on the underside of buckthorn leaf buds during the winter, and spread to soybean or its wild relatives starting in late spring. Under favorable conditions, once soybean aphids colonize on susceptible plants in early summer, their populations can grow at an exponential rate and build to thousands of aphids per soybean plant [6–8].

Spraying insecticides is the primary method of controlling soybean aphid in infested soybean fields, though it involves significant costs and environmental concerns. There was a 130-fold increase in the use of insecticides in the northcentral United States from 2000 to 2006, mainly to control soybean aphids [9]. Intensive use of insecticides may lead to insecticide resistant aphids [10, 11]. Also, beneficial insect populations can be negatively affected due to intensive uses of chemicals [12, 13].

Genetic host resistance is an alternative, cost-effective and environmentally sustainable strategy to protect soybean crops from the aphids [14]. A number of soybean aphid resistant germplasms were identified through large scale screenings against different biotypes of soybean aphids [6, 15]. Several resistance (*Rag*) genes and quantitative trait loci (QTL) with resistance to *A. glycines* have been mapped and aphid resistant cultivars adapted to the northcentral United States have been released [16–22]. A major gene was mapped on chromosome 13 in PI 567301B at a genomic position near the *Rag*2 locus in PI 243540 and provisionally named *Rag*5 [22]. This gene explained over 80% of the phenotypic variance of resistance to *A. glycines* in PI 567301B [22]. Although the two genes were mapped in close proximity, the mechanisms of resistance of the two genes were different. Detached leaves of PI 567301B lost resistance to soybean aphids, while those of PI 243540 maintained resistance [23]. *Rag*5 confers antixenosis resistance to soybean aphids, while *Rag*2 resistance is predominantly antibiosis [22].

The influence of phloem feeding insects, such as aphids, has been well studied in many plant species, including *Arabidopsis*, maize, tomato, celery, and sorghum [24–28]. Soybean-*A. glycines* interaction was also studied by employing genotypes conferring *Rag*1 or *Rag*2 resistance [29–31]. Transcriptomic studies have revealed continuity in the molecular responses to aphids across the plant species, where the expression of genes associated with cell wall development, defense, transcription factors, DNA/RNA processing, secondary metabolism, and hormone-related signaling were altered [32].

RNA sequencing enables profiling of genome-wide changes in gene expression over times under the conditions of interest. This study aimed to investigate global changes in gene expression of homozygous resistant (*Rag*5/*Rag*5) and homozygous susceptible (*rag*5/*rag*5) NILs of soybean infested by soybean aphid biotype 2. Results of the study provide insights into the interactions between soybean and *A. glycines* and identify transcriptomic changes in soybean that may be associated with resistance to *A. glycines*.

## Results and discussion

### High throughput sequencing and validation of selected DEGs by quantitative RT-PCR

A total of 24 libraries were sequenced on three lanes of the HiSeq2000. Approximately 386 million of the preprocessed reads were aligned to the latest soybean reference genome Glyma.Wm82.a2.v1, with overall mapping rate of 90% of unique reads (mapped/filtered) (Table 1). Variations by lane were adjusted via estimated size factors for data matrix using DESeq2 [33]. After the adjustment, the replicates were generally clustered based on principal component analysis (Additional file 1: Fig. S1). Of the 56,054 soybean annotated reference genes, a total of 35,284 genes were detected as ‘expressed’ with a minimum of 10 reads across the 24 samples. Some housekeeping or reference genes [34, 35] were confirmed with no differences in their expression between the R- and S-NILs throughout the four time points, including *Actin* (Glyma.08G182200), *Cons*4 (Glyma.12G020500), *Cons*6 (Glyma.12G051100), and *Tublin* (Glyma.08G014200) (Additional file 2: Table S1).

**Table 1:** Numbers of reads sequenced and mapped per sample

Genotype	Hour after infestation	Biological replicates	Total read (millions)	Read filtered (millions)	Read mapped (millions)	Percentage of mapped/filtered
R-NIL (*Rag*5/*Rag*5)	0	1	8.7	8.6	7.7	90
	0	2	21.4	21.0	19.0	90
	0	3	21.2	20.8	18.9	91
	6	1	6.9	6.8	6.2	90
	6	2	17.9	17.5	15.8	91
	6	3	19.5	19.0	16.8	88
	12	1	5.5	5.5	5.0	91
	12	2	19.7	19.3	17.4	90
	12	3	21.6	21.1	19.2	91
	48	1	8.0	7.8	6.5	83
	48	2	24.8	24.3	22.0	90
	48	3	26.6	26.0	23.2	89
S-NIL (*rag*5/*rag*5)	0	1	9.5	9.3	8.4	90
	0	2	28.7	28.1	24.8	88
	0	3	28.1	27.4	24.7	90
	6	1	10.8	10.6	9.6	90
	6	2	24.7	24.1	21.7	90
	6	3	29.2	28.5	25.8	90
	12	1	7.9	7.7	7.0	90
	12	2	13.9	13.6	12.3	90
	12	3	21.9	21.5	19.5	91
	48	1	7.9	7.8	7.0	90
	48	2	26.0	25.5	22.9	90
	48	3	27.5	26.9	24.4	91
Total			437.9	428.7	385.8	90

**Fig. 1:**
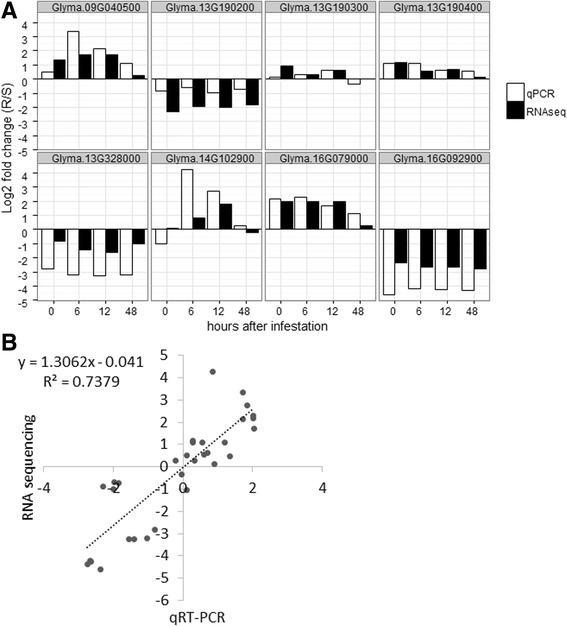
**a** Validation of results by RNA sequencing using quantitative RT-PCR. **a**, Relative expression levels were measured by qRT-PCR and compared to those of RNA sequencing for 8 selective genes. **b** Correlation in log_2_fold change (R/S) between RNA sequencing and qRT-PCR

To test the robustness of the RNA sequencing data, the expression levels of eight DEGs were validated by quantitative RT-PCR (qRT-PCR). The validation was carried out using independent samples not previously tested by RNA sequencing. Further, five of these eight genes are annotated to have resistance-related functions, and three genes (Glyma.13G190200, Glyma.13G190300, and Glyma.13G190400) are located near the *Rag*5 locus (Additional file 3: Table S2). Figure 1a shows relative expression level – log_2_fold change (R-NIL/S-NIL) – of each gene in RNA sequencing and in qRT-PCR at each time point. In most cases, the direction of differential expression in both techniques was consistent, though there were some differences in magnitude of expression between the techniques. Nonetheless, these genes showed good agreement (*R* ^2^ = 0.74) between the RNA sequencing and qRT-PCR results overall (Fig. 1b), indicating good reproducibility of the experiment.

### Substantial transcriptomic changes between pre- and post-infestation in R- or S-NILs

**Fig. 2:**
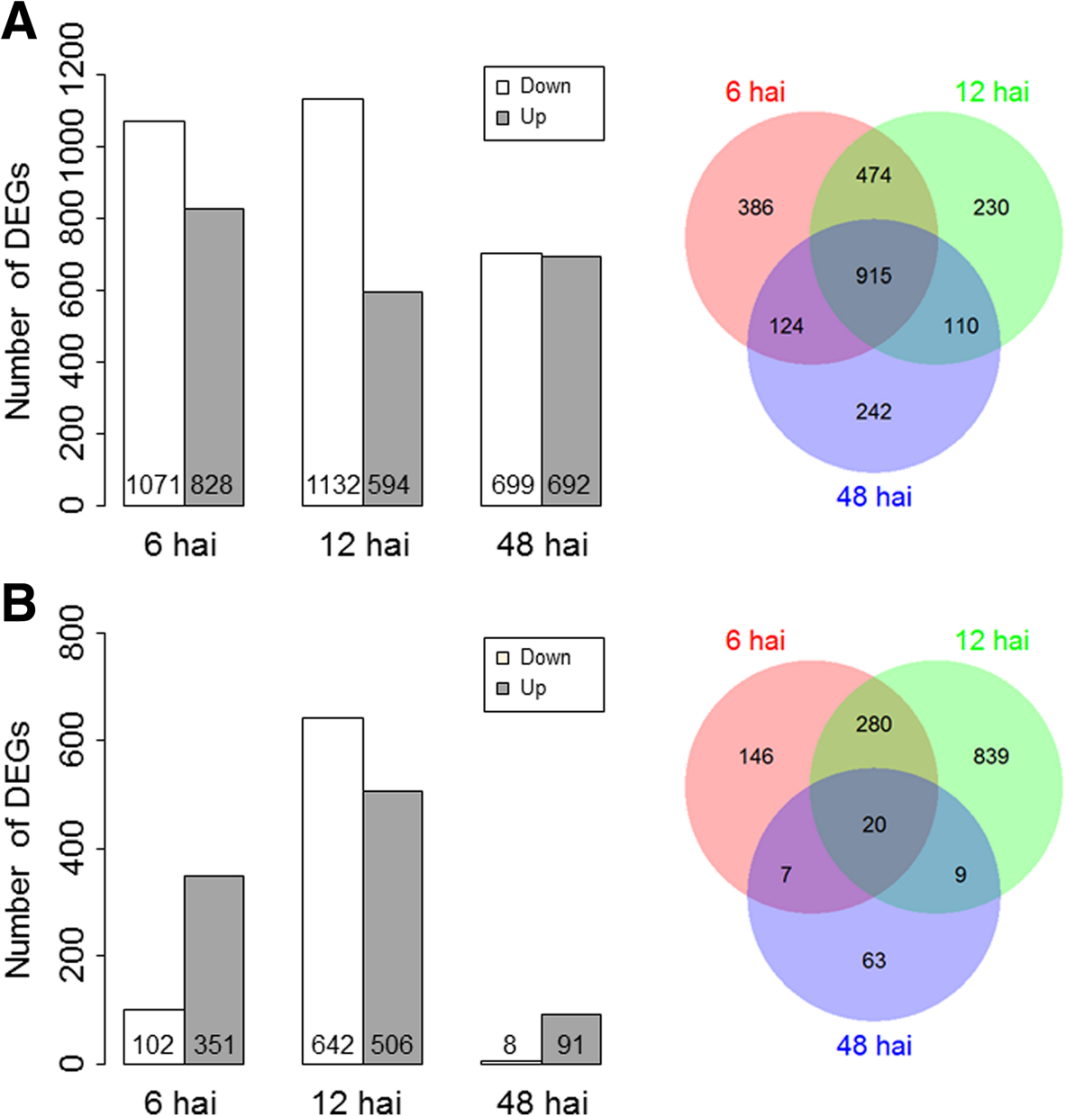
Counts of differentially expressed genes (DEGs) in the comparison between pre- and post-infestation in the R-NIL **a** and S-NIL **b** filtered with false discovery rate (FDR) < 0.01, |log2fold change (post−/pre-infestation)| > 2, and average read count >30

The post-infestation (6, 12, and 48 hai) gene expression data of the R- and S-NIL were compared with the pre-infestation control (time 0) of the respective NIL. A total of 2481 and 1364 genes were up- or down-regulated in the R- and S-NIL, respectively, in response to infestation by the aphids (Fig. 2a, b). In the R-NIL, 1899 genes were altered within 6 hai, with the numbers of DEGs gradually decreasing to 1391 by 48 hai (Fig. 2a). In the S-NIL, on the other hand, changes in gene expression were less extensive and confined primarily to 12 hai (Fig. 2b). A total of 1148 DEGs were identified at 12 hai, whereas only 453 and 99 DEGs were identified at 6 and 48 hai, respectively (Fig. 2b). The number of DEGs consistent over two-day period (6, 12, and 48 hai) largely differed between the R- and S-NIL; 915 (434 up-and 81 down-regulated) and 20 (all up-regulated) DEGs in the R- and S-NILs, respectively. Sixteen DEGs were common between the 915 DEGs of R-NIL and the 20 DEGs of S-NIL. Regardless of NIL, such DEGs included genes associated with phytohormone signaling and defense response to plant (Additional files 4 and 5: Tables S3 and S4). This suggests the mechanisms of aphid infestation response was, in part, shared between the NILs but more pronounced in the R-NIL.

At 6 and 12 hai, 2239 and 1301 DEGs were identified in the respective R- and S-NIL (Fig. 2), of which 1044 DEGs were common in both NILs and 2496 DEGs were specific to R- or S-NIL. Enrichment of gene ontology (GO) terms was analyzed separately by the group of the DEGs, and significant GO terms identified at false discovery rate (FDR) < 0.05 are listed in Table 2. A total of 24 significant GO terms were identified in the three groups. Functional analysis of GO terms indicates that soybean responses to aphid infestation agree with current understanding in mechanisms of plant defense in various hosts – aphid systems [26, 29, 36, 37]. Carbohydrate metabolic process (GO:0005975) and oxidation reduction (GO:0055114) were most highly enriched in the common DEGs significant in both compatible and incompatible interaction of the present study (Table 2). An evolutionary arm race, called the Zig-zag model [38], was proposed to illustrate the molecular interaction between plant hosts and parasites. According to the model, plants detect pathogen-associated molecular patterns (PAMPs), such as chitin and flagellin, and trigger PAMP-associated immunity (PTI). Successful pathogens interfere PTI by secreting effectors to manipulate host cell process. Effectors may trigger susceptibility in plants. Once effectors are recognized by nucleotide binding site-leucine-rich repeat (NBS-LRR) protein, effector-triggered immunity (ETI) is activated. Aphid stylets and exoskeleton are composed of chitin, same as fungal PAMPs [39]. Although it is yet unclear if the Zig-zag model [38] can be extended to aphids or other insects, plants may recognize aphids’ stylets or components in aphid saliva and turn on PAMP-triggered immunity (PTI). PTI can initiate chemical defense, such as generation of reactive oxygen species (ROS), and mechanical defense by cell wall modification and deposition of callose [25, 40]. ROS is involved in defensive signaling in plant tissues, and plants also induce detoxifying proteins that detoxify these oxidative stress [32]. Genes involved in hydrogen peroxide biosynthetic process such as Glyma.09G038500, Glyma.06G225500, and Glyma.04G153700 were found highly upregulated (>8 times) in the R-NIL after aphid infestation (Additional file 4: Table S3), while upregulation was delayed and limited at 12 hai in S-NIL. Related to detoxification, oxidoreductase activity (e.g. GO:0016701, GO:0016491, GO:0016703, GO:0016903) was also found statistically significant in both R- and S-NILs. Genes associated with fatty acid biosynthetic process (GO:0006633) and fatty acid metabolic process (GO:0006631), lipid metabolic process (GO:0006629) were enriched in R-specific response. This may be related because plants are known to consume polyunsaturated fatty acids (linoleic and linolenic acid) for jasmonic acid (JA) biosynthesis [31]. Activated JA signaling was effective to significantly reduce aphids in *Arabidopsis* [41]. Some genes related to JA biosynthetic process such as Glyma.07G034800, Glyma.15G026400, Glyma.14G103100, and Glyma.19G008700 were highly upregulated (averaging over 5-folds) in R-NIL after aphid infestation (Additional file 4: Table S3). Hormone-related signaling triggered by aphid infestation including JA, salicylic acid, and ethylene, plays critical roles in regulating plant defenses [32].

**Table 2:** Enriched gene ontology terms identified from the 2496 differentially expressed genes in the R- and/or S-NILs during 12 h after aphid infestation

Class	GO accession	Term type^a^	Term description	Query item	Query total	Background item	Background total	FDR
Common	GO:0005975	P	Carbohydrate metabolic process	61	622	1443	29,501	0.000
	GO:0055114	P	Oxidation reduction	80	622	2408	29,501	0.018
	GO:0016052	P	Carbohydrate catabolic process	14	622	195	29,501	0.028
	GO:0016701	F	Oxidoreductase activity, acting on single donors with incorporation of molecular oxygen	14	622	108	29,501	0.000
	GO:0016491	F	Oxidoreductase activity	98	622	2744	29,501	0.000
	GO:0016703	F	Oxidoreductase activity, acting on single donors with incorporation of molecular oxygen, incorporation of one atom of oxygen (internal monooxygenases or internal mixed function oxidases)	6	622	11	29,501	0.000
	GO:0010277	F	Chlorophyllide a oxygenase activity	6	622	11	29,501	0.000
	GO:0003682	F	Chromatin binding	32	622	605	29,501	0.001
	GO:0051537	F	2 iron, 2 sulfur cluster binding	6	622	18	29,501	0.001
	GO:0016798	F	Hydrolase activity, acting on glycosyl bonds	34	622	759	29,501	0.005
	GO:0004497	F	Monooxygenase activity	8	622	64	29,501	0.007
	GO:0004553	F	Hydrolase activity, hydrolyzing O-glycosyl compounds	32	622	726	29,501	0.007
	GO:0051536	F	Iron-sulfur cluster binding	10	622	102	29,501	0.007
	GO:0051540	F	Metal cluster binding	10	622	102	29,501	0.007
	GO:0016903	F	Oxidoreductase activity, acting on the aldehyde or oxo group of donors	8	622	66	29,501	0.008
	GO:0004650	F	Polygalacturonase activity	9	622	112	29,501	0.045
Common	GO:0034357	C	Photosynthetic membrane	11	622	117	29,501	0.004
	GO:0009521	C	Photosystem	11	622	113	29,501	0.004
	GO:0009579	C	Thylakoid	11	622	126	29,501	0.005
	GO:0016020	C	Membrane	103	622	3518	29,501	0.010
	GO:0044436	C	Thylakoid part	7	622	59	29,501	0.010
	GO:0044425	C	Membrane part	59	622	1801	29,501	0.013
	GO:0019898	C	Extrinsic to membrane	6	622	50	29,501	0.016
	GO:0009522	C	Photosystem I	5	622	35	29,501	0.018
	GO:0009654	C	Oxygen evolving complex	5	622	42	29,501	0.034
	GO:0031224	C	Intrinsic to membrane	47	622	1453	29,501	0.035
R-specific	GO:0055114	P	Oxidation reduction	93	740	2408	29,501	0.009
	GO:0006629	P	Lipid metabolic process	49	740	1024	29,501	0.009
	GO:0006633	P	Fatty acid biosynthetic process	12	740	108	29,501	0.009
	GO:0006631	P	Fatty acid metabolic process	13	740	130	29,501	0.009
	GO:0032787	P	Monocarboxylic acid metabolic process	15	740	176	29,501	0.011
	GO:0043436	P	Oxoacid metabolic process	31	740	580	29,501	0.011
	GO:0006082	P	Organic acid metabolic process	31	740	581	29,501	0.011
	GO:0019752	P	Carboxylic acid metabolic process	31	740	580	29,501	0.011
	GO:0042180	P	Cellular ketone metabolic process	31	740	584	29,501	0.011
	GO:0008610	P	Lipid biosynthetic process	25	740	470	29,501	0.040
	GO:0008152	P	Metabolic process	401	740	14,225	29,501	0.046
	GO:0016491	F	Oxidoreductase activity	103	740	2744	29,501	0.023
S-specific	GO:0015979	P	Photosynthesis	6	149	143	29,501	0.029
	GO:0034357	C	Photosynthetic membrane	6	149	117	29,501	0.001
	GO:0009521	C	Photosystem	6	149	113	29,501	0.001
	GO:0009579	C	Thylakoid	6	149	126	29,501	0.001

^a^P, biological process; F, molecular function; C, cellular component

At 48 hai, 1391 DEGs were identified in the R-NIL, while only 99 DEGs were in the S-NIL (Fig. 2a, b). Of those in the R-NIL, 242 genes were unique at 48 hai (Fig. 2a). These DEGs generally differed in their functions from those responded earlier at 6 and 12 hai. The frequencies of DEGs involved in DNA replication (GO:0006260, FDR = 2.7e-18), DNA metabolism (GO:0006259, FDR = 4.4e-10), and ligase activity (GO:0051002, FDR = 4.6e-5) were highly significant in enriched GO term analysis (Additional file 6: Table S5). This was different from the S-NIL because such GO terms were not significant or remarkable from 63 DEGs unique at 48 hai in the S-NIL (Additional file 7: Table S6). This may be related to aphid resistance in R-NIL, because the R-NIL may immediately begin to recover damaged cells or regenerate new cells after the initial interaction with aphids. In contrast, these processes may not occur immediately or be delayed in the susceptible line.

### Comparison of mRNA abundance between R- and S-NILs following aphid infestation

**Fig. 3:**
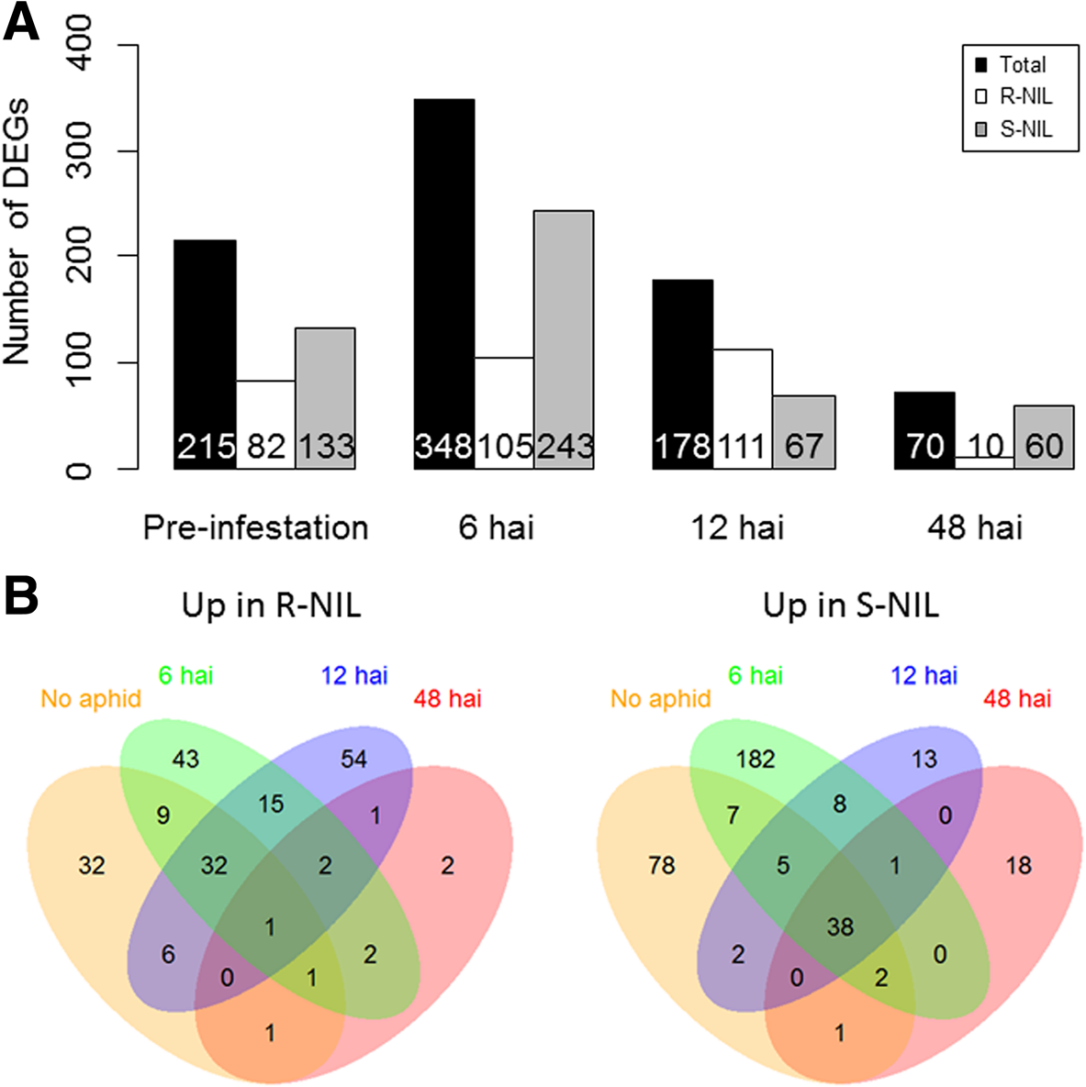
**a** Counts of differentially expressed genes (DEGs) identified in the comparison between R- and S-NILs at pre-infestation, 6, 12, and 48 h after infestation, filtered with false discovery rate (FDR) < 0.1, |log2fold change (R/S)| > 1, and average read count >10. R-NIL indicates the number of DEGs upregulated in R-NIL, while S-NIL does those upregulated in S-NIL at each time point. **b** Venn diagram displaying the detailed distribution of DEGs

While comparison between pre- and post-aphid infestation explained general aspects of transcriptomic reprograming in response to aphid infestation, changes in abundance of transcripts between the R- and S-NILs for different time points would provide a better understanding of regulation of *Rag*5-conferring soybean aphid resistance in PI 567301B. Relaxed stringency criteria – FDR < 0.1, |log_2_fold change| > 1, and average read >10 – were adopted for this comparison between R- and S-NILs since the same stringency as pre- vs. post-infestation resulted in a very small number of the DEGs. A total of 556 DEGs were identified between the R- and S-NILs, which were statistically significant at one or more time points (Additional file 8: Table S7). The resistant and susceptible genotypes are backcross (BC_5_) lines, and thus highly (>98%) genetically identical to the recurrent parent (i.e. Wyandot). Thus, while the number of DEGs between NILs is small, they are more likely to reflect biological significance rather than genetic background. Dynamic changes in the gene expression level between the two genotypes were observed during the initial 12 h, and the total number of DEGs was considerably smaller at 48 hai (Fig. 3). This result suggests that the initial 12 h is a critical period in soybean-aphid interaction, supporting the earlier reports that approximately 8–9 hai was the key time period of soybean-aphid interaction [29]. In the present study, transcripts of 76 genes were consistently and significantly up-regulated in one NIL than the other for at least 12 hai. These genes are listed in Additional file 9: Table S8. Not surprisingly, the annotated functions of these genes were mostly similar with the defense mechanisms found in the comparison of pre- vs. post-infestation. GO term enrichment analysis of the 76 DEGs identified only significant GO term for ADP binding (GO:0043531). This GO term is far too generalized to make any inference regarding underlying mechanism. Corresponding DEGs are related to defense response, signal transduction, and SA biosynthetic process (Additional file 9: Table S8).

Aphid behaviors such as probing and feeding can be affected by biochemical components such as pH, sucrose, and amino acid contents in phloem sap [40, 42]. This can be one reason of preference leading to antixenosis type of resistance provided by the *Rag*5 gene. This interaction would be likely determined within a short period of time after initial contact. Thus, a chemical component would more likely be constitutive in resistant genotype. Similarly, in transcript levels, constitutively expressed genes were thought to be responsible for many defense-related responses or differential priming of resistance in resistant plants, including wheat [43]. Studham and MacIntosh [31] also discussed that constitutive resistance might be, in part, responsible for the response observed in R NILs under aphid-absent condition. In the present study, 82 DEGs were upregulated in the R-NIL compared to the S-NIL in the absence of aphids, and 14 of these genes are annotated as leucine-rich repeat (LRR)-containing protein or LRR receptor-like protein kinase. These proteins are related to defense response [44] and may confer the ability to rapidly recognize aphid infestation and trigger initial responses. Many secondary metabolites, such as phenylpropanoids and terpenoids, have been implicated in plant defense against phloem-feeding insects [32], yet unknown if these DEGs are connected to metabolomic differences in the NILs. Elucidating this relationship could provide further understanding of antixenosis.

### Thirteen candidate genes located in the *Rag*5 locus of chromosome 13

After initial genetic mapping of the *Rag*5 gene in the Wyandot × PI 567301B population, backcross populations developed using Wyandot as the recurrent parent were used to fine map the *Rag*5 locus. The locus was placed within a 120-kb interval (Gm13:30.33 ~ 30.35 Mb) on chromosome 13 (unpublished data). The published soybean genome was sequenced using the aphid-susceptible variety Williams 82 [45] that does not have the *Rag*5 allele. Williams 82 has 13 annotated genes in the 120-kb interval containing the *Rag*5 locus (Table 3). There are three copies of genes with the annotated function ‘protease family S26 mitochondrial inner membrane protease’ (Table 3). One of them, Glyma.13G190200, showed a significant difference in gene expression (FDR < 0.001) and was expressed approximately 4-fold higher in S-NIL for all time points (Table 3). Another ‘protease family S26 mitochondrial inner membrane protease’ gene (Glyma.13G190500) was also significant at 6 (FDR < 0.05) and 12 hai (FDR < 0.001) with almost 4-fold higher expression in R-NIL. It is unknown why the gene encoding ‘protease family S26 mitochondrial inner membrane protease’ is related to aphid resistance and even why the two genes with the same annotation responded differentially during the two-day period. A gene of unknown function (Glyma.13G190600) was also detected with four-fold higher mRNA expression in R-NIL than in S-NIL, which was consistent from 0 to 12 hai (Table 3).

**Table 3:** Thirteen genes annotated in the *Rag*5 candidate region of chromosome 13 based on Glyma.Wm82.a2.v1 and comparison of their transcript abundance between R- and S-NIL

Gene ID	Annotation	Log2fold change (R/S)^a^			
		0 hai	6 hai	12 hai	48hai
Glyma.13g189800	Member of ‘GDXG’ family of lipolytic enzyme	0.4	1.5**	0.7	0.7
Glyma.13g189900	Choline/Ethanoalamine kinase	0.1	0	0	0
Glyma.13g190000	Leucine-rich repeat-containing protein	0.6	-0.5	-0.2	-0.3
Glyma.13g190100	ORF protein	-0.2	0	0	0.4
Glyma.13g190200	Protease family S26 Mitochondrial inner membrane protease	-2.3****	-2.0****	-2.0****	-1.8***
Glyma.13g190300	Leucine-rich repeat-containing protein	0.9	0.3	0.6	0
Glyma.13g190400	Leucine-rich repeat-containing protein	1.2	0.5	0.7	0.1
Glyma.13g190500	Protease family S26 Mitochondrial inner membrane protease	1.3	1.7*	2.1****	0.8
Glyma.13g190600	Present only in the Glyma.Wm82.a2 assembly	2.2****	2.1****	2.0****	0.6
Glyma.13g190700	Present only in the Glyma.Wm82.a2 assembly	0.1	0.1	0	0
Glyma.13g190800	Leucine-rich repeat-containing protein	1.1	0.7	0.9	0.2
Glyma.13g190900	Protease family S26 Mitochondrial inner membrane protease	0.2	0.3	0.5	0.1
Glyma.13g191000	Protein of unknown function	0	0.1	0.1	0.4

^a^Asterisks indicate significance levels: *, FDR < 0.05; **, FDR < 0.01; ***, FDR < 0.005; ****, FDR < 0.001

NBS-LRR genes are well known as a major group of genes conferring resistance against virulent biological agents in many crops [44, 46]. The first cloned root knot nematode resistance gene in tomato, *Mi-1.2*, is an NBS-LRR type of plant resistance gene [47]. Aphid-resistance genes identified in melon and *Medicago truncatula* were mapped to a NBS-LRR gene cluster [48, 49]. In the soybean – *A. glycines* interaction, microarray contrasting *Rag*1 genotype (cv. Dowling) and susceptible genotype (Williams 82) also identified an NBS-LRR type of gene with constitutively higher expression during 3 days after aphid feeding, which was proposed as a strong candidate gene for *Rag*1 [29]. Recently, transcriptomic profiling using the NILs carrying either *Rag*2 or *rag*2 allele from PI 243540 revealed that a NBS-LRR coding gene, Glyma13g25970 (Glyma.13 g190400 in Glyma.Wm82.a2.v1), located in the *Rag*2 (PI 243540) interval (unpublished) was highly upregulated in the resistant *Rag*2 (PI 243540)-NIL [30]. Also, two non-NBS-LRR coding genes, Glyma13g25990 (no correspondence in Glyma.Wm82.a2.v1) and Glyma13g26010 (Glyma.13 g190200 in Glyma.Wm82.a2.v1), in the *Rag*2 (PI 200538) interval were found significantly upregulated in the resistant *Rag*2-NIL [30]. It was reported that PI 243540 and PI 567301B have distinct modes of resistance against biotype 2 aphid [23]. Different genes may be involved in the two modes of resistance and thus, regulation of Glyma.13G190200 is not necessarily same in the two modes of resistance.

The fine mapped genomic interval of *Rag*5 partly overlapped with the (~90 kb) window of *Rag*2 (PI 243540) (unpublished data) and with the candidate genomic region (54 kb) of *Rag*2 (PI 200538) [50]. This region is a well-known *R*-gene clustered region. Among the thirteen genes, four were predicted to encode LRR-containing protein (Glyma.13 g190000, Glyma.13 g190300, Glyma.13 g190400, and Glyma.13 g190800). Two genes (Glyma.13 g190400 and Glyma.13 g190800) showed two-fold higher expression in the R-NIL only at no aphid infestation (time 0), while the FDR of the respective genes was greater than the filtering threshold of 0.1. However, the difference between R- and S-NILs for both genes diminished after aphid infestation (Table 3). This result suggests that such LRR type of genes may not be primarily responsible for *Rag*5-conferring aphid resistance. With different modes of resistance of *Rag*2 (antibiosis) and *Rag*5 (antixenosis), it is expected that the molecular mechanisms of resistance of the two genes will be different, even though their genomic locations may be in a close proximity. It is also plausible that our study may not be able to capture the key genes in this region, because the genome of Williams 82 does not possess any aphid resistance allele.

## Conclusions

The present study compared changes in mRNA abundance following aphid infestation of resistant and susceptible NILs developed for the *Rag*5 locus. Many DEGs were common between the two genotypes and were functionally relevant to known defense mechanisms reported in various host-aphid systems. The responses to aphid infestation in the two NILs significantly differed in timing of changes in gene regulation, which was triggered more quickly in the R-NIL. There was also a high level of commonality found in the DEGs between the NILs, which mostly function in mediating plant defense against aphids. Transcriptomic analyses on R- and S-NILs provided reduced lists of DEGs at each time point when compared to studies conducted with unrelated pair of genotypes.

By assessing the DEGs in the *Rag5*-containing QTL region on chromosome 13, three non-NBS-LRR candidate genes – Glyma13g190200, Glyma13g190500, and Glyma13g190600 –showed distinct changes in their expression between R- and S- NILs. Moreover, four NBS-LRR genes in the candidate region did not have differential expression between R- and S-NILs at any time point. Our results indicate that the *Rag*2 and *Rag*5 resistance may be conditioned by different genes. Further studies, such as metabolomics and proteomics, will be helpful to examine the roles of these candidates in aphid resistance.

## Methods

### Plant materials, aphid infestation, and sampling

PI 567301B was identified as a source of Rag5 by QTL mapping with Wyandot x PI 567301B RIL population [22]. For fine-mapping of the QTL and further studies, NILs segregating at the resistance locus *Rag*5 were developed at USDA-ARS, Wooster, OH by five backcrosses (BC) using the cultivar Wyandot as the recurrent parent and PI 567301B as the donor parent. During development and selection of BC lines, the soybean aphid resistance of each BC line and two parental lines was evaluated by counting the number of aphid per plant at 3 weeks after infestation using the method published earlier [22]. A plant was scored as resistant if it had <25 aphids and a plant was scored as susceptible if it had >500 aphids. There was a clear difference in the aphid counts to identify a resistant plant from a susceptible one. As a precaution, plants with aphid numbers between 25 and 500 were discarded. All plants were also screened with 10 SSR markers between OSU180 and BARCSOYSSR_13_1168, flanking the *Rag*5 locus [22]. BC lines were selected based on quality of phenotypic data and marker genotypes and two BC_5_ NILs (R- and S-NILs) were finally selected in the fall of 2010 from a total of 120 BC_5_F_3_ plants segregating for the *Rag*5 locus. Individual seed of homozygous aphid resistant (*Rag*5/*Rag*5) and susceptible (*rag*5/*rag5*) BC_5_ lines were planted in 4-cm diameter × 15-cm deep plastic cone-tainers (Stuewe & Sons, Tangent, OR) and seedlings were grown in a growth chamber in OARDC. The growth chamber was maintained at 24/20 °C and 16 h/8 h day/night temperatures and light/dark periods, respectively.

Biotype 2 aphid was first collected in a soybean field at OARDC, Wooster, OH, which was confirmed as a new biotype of soybean aphid and named as biotype 2 [51]. A pure laboratory colony of biotype 2 has been maintained in a growth chamber at OARDC and all aphids used in this experiment were collected from this growth chamber colony. Fifteen adult aphids were placed on the top leaves of resistant and susceptible plants at V2 stage. The youngest partially expanded trifoliate leaf along with the growing point from each seedling was sampled in a 2-ml tube at time 0 (i.e., without aphid infestation), and at 6, 12, and 48 hai. Each seedling was sampled once and discarded immediately. At each collection time, a new set seedlings from the remaining infested seedlings were used for tissue collection. The tissue from each seedling represented a replicate in the study. At tissue collection at 6 and 12 hai, more aphids were added on seedlings meant for later time points if less than 10 aphids were counted on a seedling, in order to maintain continuous aphid pressure on each seedling. All aphids and nymphs were removed from the leaf before tissue collection. For time 0 samples, leaves were collected just before infestation of aphids. Leaf samples were immediately frozen in liquid nitrogen and later at −80 °C.

### RNA preparation and high-throughput sequencing

Collected leaf tissue was ground by a pestle in liquid nitrogen and total RNA was extracted using RNeasy Plant Mini Kit (Qiagen, Germantown, MD, USA) according to the manufacturer’s instructions. Quality and quantity of the extracted RNA was assessed using Agilent RNA 6000 Nano Kit and 2100 Bioanalyzer (Agilent Technologies, Santa Clara, CA, USA). RNA (1μg/sample) was used to synthesize a double-stranded (ds) cDNA library for each sample using Illumina’s TruSeq RNA Library Prep Kit v2 (Illumina Inc., San Diego, CA, USA) as per the manufacturer’s instructions. The ds-cDNA libraries were quantified using the Qubit 2.0 fluorometer (Life Technologies, Carlsbad, CA, USA) and quality of each library was assessed using Agilent DNA 1000 Kit and 2100 Bioanalyzer (Agilent Technologies, Santa Clara, CA, USA). Of the total 24 cDNA libraries (4 time points × 2 genotypes × 3 replicates), 8 libraries representing single replicates were multiplexed with different barcode sequences embedded in preparation for high-throughput sequencing. Each of the multiplexed libraries (50 mol) was sequenced in 100-bp paired-end fashion, in three lanes of Illumina HiSeq2000 platform at the Ohio State University’s Comprehensive Cancer Center, Columbus, OH, USA.

### Read mapping and statistical analyses

The Galaxy platform facilitated the execution of software for analyzing high-throughput sequencing data, which is maintained by the Molecular and Cellular Imaging Center Computational Biology Laboratory (MCBL) at OARDC. *FastQC* [52] was used to obtain statistics on read quantity and quality before preprocessing. *Cutadapt* [53] was used to remove adapter sequences and poly-A and -T tails with default settings except the followings; 3′ end and minimum overlap length of 6. Then, read sequences were controlled by the function *trim the reads by quality* using the default options with the following changes; 40 for length threshold, 20 for quality threshold (Phred score), and discard of internal *N*.

Read mapping to the soybean genome assembly (Glyma.Wm82.a2.v1) was conducted by the splice junction mapper, Tophat2 [54], with the default options except; 50 for mean inner distance between mate pairs, 100 for standard deviation for distance between mate pairs, and no report discordant pair alignment. A few input parameters were changed as followings; minimum intron length 70 and maximum intron length 20,000. The reads mapped to the genome were counted using HTSeq [55] and the mode *union* was used to handle multi-mapping reads. The quantified transcript read files were imported into Bioconductor, an open-source software project based on the R programming language [56]. Effects of aphid infestation were initially investigated by comparing changes in gene expression between pre- and post-aphid infestation in the respective R- and S-NILs using DESeq2 [33]. DEGs were filtered as the following criteria; mean read counts >30, |log_2_fold change| > 2, adjusted *P*-value (false discovery rate, FDR) < 0.01. Transcriptomic differences caused by aphid infestation between R- and S-NILs were subsequently identified at each time point and the DEGs were filtered by thresholds - mean read counts >10; |log_2_fold change| > 1; FDR < 0.1.

### Gene ontology (GO)

GO term enrichment analysis on the various groups of DEGs was conducted using agriGO (available at http://bioinfo.cau.edu.cn/agriGO/) for Singular Enrichment Analysis [57]. For each analysis, *Glycine max v2.0* was selected as the species and *Glycine max Wm82.a2. v1* was used as reference with defaults in other parameters, including FDR < 0.05.

### Validation of gene expression using quantitative reverse transcription (qRT)-PCR

cDNA was transcribed using Bio-Rad® iScript synthesis kit (Bio-Rad, Hercules,CA). Primer pairs were designed to confirm expression of 8 targeted genes using Primer 3 [58], as listed in Additional file 3: Table S2. *Actin* (Gyma.08G182200) [34, 35] was used as the endogenous qRT-PCR control using forward primer 5′-AGTTCTGCAGTGGAAAAGAGCTA-3′ and reverse primer 5′-TCATGAATTCCAGTAGCTTCCAT-3′. Real-time quantification was conducted using Bio-Rad® iScript iQ™ SYBR Green Supermix Kit and Bio-Rad® CFX96™ system. Final volume 15ul of PCR mixture included 200 ng of cDNA, 400 nM of each primer, 2.7ul of sterile water and 7.5ul of iQ SYBR Green Supermix. PCR amplification was conducted as manufacturer’s suggestion as the following: 95 °C for 3 min, 35 cycles of 95 °C for 10s and 59 °C for 30s, and followed by melt curve analysis. There were 3 biological replications and 2 technical replications for each gene per time point. Relative expression levels and fold changes were determined using comparative *C*_T_ method [59]. Statistical analysis was conducted using PROC TTEST in SAS 9.4 (SAS institute, Cary, NC).

## Additional files

Additional file 1: Figure S1. Principle component analysis of 24 cDNA library samples. (TIFF 46 kb)

Additional file 2: Table S1. Transcriptomic changes in four reference genes between the R-NIL and S-NIL. (XLSX 9 kb)

Additional file 3: Table S2. Primer sequences for quantitative RT-PCR for validating RNA sequencing data of selected eight genes. (XLSX 10 kb)

Additional file 4: Table S3. Gene ontology and relative transcript abundance of the 915 DEGs consistently significant in the R-NIL. (XLSX 114 kb)

Additional file 5: Table S4. Gene ontology and relative transcript abundance of the 20 DEGs consistently significant in the S-NIL. (XLSX 14 kb)

Additional file 6: Table S5. Enriched gene ontology terms identified from the 242 differentially expressed genes in the R-NIL at 48 h after aphid infestation. (XLSX 24 kb)

Additional file 7: Table S6. Enriched gene ontology terms identified from the 63 differentially expressed genes in the S-NIL at 48 h after aphid infestation. (XLSX 50 kb)

Additional file 8: Table S7. Number of reads and normalized read counts of the 556 DEGs from comparison between the R-NIL and the S-NIL. (XLSX 270 kb)

Additional file 9: Table S8. Gene ontology of 76 DEGs consistently up-regulated during 12 hai or later either in the R- or the S-NIL. (XLSX 22 kb)

## Abbreviations

DEG: Differentially expressed gene
FDR: False discovery rate
GO: Gene ontology
NBS-LRR: Nucleotide binding site-leucine rich repeat
qRT-PCR: Quantitative reverse transcription-polymerase chain reaction
*Rag*: Resistance to *Aphis glycines*

## References

[1] Wang S, Bao X, Sun Y, Chen R, Zhai B (1996). Study on effect on population dynamics of soybean aphid (*Aphis glycines*) on both growth and yield of soybean. Soybean Sci.

[2] Hartman GL, Domier LL, Wax LM, Helm CG, Onstad DW, Shaw JT, et al. Occurance and distribution of *Aphis glycines* on soybeans in Illinois in 2000 and its potential control. Plant Health Prog. 2001; doi:10.1094/PHP-2001-0205-01-HN.

[3] Venette RC, Ragsdale DW. Assessing the invasion by soybean aphid (Homoptera: Aphididae): where will it end? Ann Entomol Soc Am. 2004;97(2):219–26. doi:10.1603/0013-8746(2004)097[0219:ATIBSA]2.0.CO;2.

[4] Ragsdale DW, Landis DA, Brodeur J, Heimpel GE, Desneux N (2011). Ecology and management of the soybean aphid in North America. Annu Rev Entomol.

[5] Ragsdale DW, Voegtlin DJ, O'Neil RJ. Soybean aphid biology in North America. Ann Entomol Soc Am. 2004;97(2):204–8. doi:10.1603/0013-8746(2004)097[0204:SABINA]2.0.CO;2.

[6] Mensah C, DiFonzo C, Nelson RL, Wang D (2005). Resistance to soybean aphid in early maturing soybean germplasm. Crop Sci.

[7] McCornack BP, Ragsdale DW, Venette RC. Demography of soybean aphid (Homoptera: Aphididae) at summer temperatures. J Econ Entomol. 2004;97(3):854–61. doi:10.1603/0022-0493(2004)097[0854:DOSAHA]2.0.CO;2. 10.1603/0022-0493(2004)097[0854:DOSAHA]2.0.CO;2 15279264

[8] Beckendorf EA, Catangui MA, Riedell WE (2008). Soybean aphid feeding injury and soybean yield, yield components, and seed composition. Agron J.

[9] Ragsdale DW, Landis DA, Brodeur J, Heimpel GE, Desneux N (2011). Ecology and Management of the Soybean Aphid in North America. Annu Rev Entomol.

[10] Puinean AM, Foster SP, Oliphant L, Denholm I, Field LM, Millar NS (2010). Amplification of a cytochrome P450 gene is associated with resistance to neonicotinoid insecticides in the aphid *Myzus persicae*. Plos Genet.

[11] Li X, Schuler MA, Berenbaum MR (2007). Molecular mechanisms of metabolic resistance to synthetic and natural xenobiotics. Annu Rev Entomol.

[12] Johnson KD, O'Neal ME, Bradshaw JD, Rice ME. Is preventative, concurrent management of the soybean aphid (Hemiptera : aphididae) and bean leaf beetle (Coleoptera: chrysomelidae) possible? J Econ Entomol. 2008;101(3):801–9. doi:10.1603/0022-0493(2008)101[801:IPCMOT]2.0.CO;2. 10.1603/0022-0493(2008)101[801:ipcmot]2.0.co;2 18613581

[13] Desneux N, Decourtye A, Delpuech J (2007). The sublethal effects of pesticides on beneficial arthropods. Annu Rev Entomol.

[14] Hill CB, Chirumamilla A, Hartman GL (2012). Resistance and virulence in the soybean-Aphis Glycines interaction. Euphytica.

[15] Hill CB, Li Y, Hartman GL (2004). Resistance to the soybean aphid in soybean germplasm. Crop Sci.

[16] Hill CB, Li Y, Hartman GL (2006). A single dominant gene for resistance to the soybean aphid in the soybean cultivar Dowling. Crop Sci.

[17] Mian MAR, Kang ST, Beil SE, Hammond RB (2008). Genetic linkage mapping of the soybean aphid resistance gene in PI 243540. Theor Appl Genet.

[18] Li Y, Hill CB, Carlson SR, Diers BW, Hartman GL (2007). Soybean aphid resistance genes in the soybean cultivars Dowling and Jackson map to linkage group M. Mol Breed.

[19] Hesler LS, Chiozza MV, O'Neal ME, MacIntosh GC, Tilmon KJ, Chandrasena DI (2013). Performance and prospects of *Rag* genes for management of soybean aphid. Entomol Exp Appl.

[20] M. McCarville, E. W. Hodgson and M. E. O'Neal. Soybean aphid-resistant soybean varieties for Iowa. Iowa State University Extension and Outreach PM 3023Ames, IA, USA 2012. Available online at: https://works.bepress.com/erin_hodgson/68/. Accessed 14 June 2016.

[21] Mian MAR, McHale LK, Michel AP, Dorrance AE. Registration of ‘Wyandot-14’ soybean with resistance to soybean aphid and powdery mildew. J Plant Reg. 2016; doi:10.3198/jpr2015.09.0059crc.

[22] Jun TH, Mian MAR, Michel AP (2012). Genetic mapping revealed two loci for soybean aphid resistance in PI 567301B. Theor Appl Genet.

[23] Michel AP, Mian MA, Davila-Olivas NH, Canas LA (2010). Detached leaf and whole plant assays for soybean aphid resistance: differential responses among resistance sources and biotypes. J Econ Entomol.

[24] Moran PJ, Thompson GA (2001). Molecular responses to aphid feeding in Arabidopsis in relation to plant defense pathways. Plant Physiol.

[25] Divol F, Vilaine F, Thibivilliers S, Amselem J, Palauqui JC, Kusiak C (2005). Systemic response to aphid infestation by *Myzus persicae* in the phloem of *Apium graveolens*. Plant Mol Biol.

[26] Coppola V, Coppola M, Rocco M, Digilio MC, D'Ambrosio C, Renzone G (2013). Transcriptomic and proteomic analysis of a compatible tomato-aphid interaction reveals a predominant salicylic acid-dependent plant response. BMC Genomics.

[27] Tzin V, Fernandez-Pozo N, Richter A, Schmelz EA, Schoettner M, Schaefer M (2015). Dynamic maize responses to aphid feeding are revealed by a time series of transcriptomic and metabolomic assays. Plant Physiol.

[28] Zhu-Salzman K, Salzman RA, Ahn JE, Koiwa H (2004). Transcriptional regulation of sorghum defense determinants against a phloem-feeding aphid. Plant Physiol.

[29] Li Y, Zou J, Li M, Bilgin DD, Vodkin LO, Hartman GL (2008). Soybean defense responses to the soybean aphid. New Phytol.

[30] Brechenmacher L, Nguyen THN, Zhang N, Jun T, Xu D, Mian MAR (2015). Identification of soybean proteins and genes differentially regulated in near isogenic lines differing in resistance to aphid infestation. J Proteome Res.

[31] Studham ME, MacIntosh GC (2013). Multiple phytohormone signals control the transcriptional response to soybean aphid infestation in susceptible and resistant soybean plants. Mol Plant-Microbe Interact.

[32] Thompson GA, Goggin FL (2006). Transcriptomics and functional genomics of plant defence induction by phloem-feeding insects. J Exp Bot.

[33] Love MI, Huber W, Anders S (2014). Moderated estimation of fold change and dispersion for RNA-seq data with DESeq2. Genome Biol.

[34] Libault M, Thibivilliers S, Bilgin DD, Radwan O, Benitez M, Clough SJ (2008). Identification of four soybean reference genes for gene expression normalization. Plant Genome.

[35] Jian B, Liu B, Bi Y, Hou W, Wu C, Han T (2008). Validation of internal control for gene expression study in soybean by quantitative real-time PCR. BMC Mol Biol.

[36] Smith CM, Boyko EV (2007). The molecular bases of plant resistance and defense responses to aphid feeding: current status. Entomol Exp Appl.

[37] Jaouannet M, Rodriguez PA, Thorpe P, Lenoir CJ, MacLeod R, Escudero-Martinez C (2014). Plant immunity in plant-aphid interactions. Front Plant Sci.

[38] Jones JD, Dangl JL (2006). The plant immune system. Nature.

[39] Uzest M, Gargani D, Dombrovsky A, Cazevieille C, Cot D, Blanc S (2010). The “acrostyle”: a newly described anatomical structure in aphid stylets. Arthropod Struct Dev.

[40] Tjallingii WF (2006). Salivary secretions by aphids interacting with proteins of phloem wound responses. J Exp Bot.

[41] Ellis C, Turner JG (2002). A conditionally fertile *coi*1 allele indicates cross-talk between plant hormone signaling pathways in *Arabidopsis thaliana* seeds and young seedlings. Planta.

[42] Will T, Furch AC, Zimmermann MR (2013). How phloem-feeding insects face the challenge of phloem-located defenses. Front Plant Sci.

[43] Han Y, Wang Y, Bi J, Yang X, Huang Y, Zhao X (2009). Constitutive and induced activities of defense-related enzymes in aphid-resistant and aphid-susceptible cultivars of wheat. J Chem Ecol.

[44] DeYoung BJ, Innes RW (2006). Plant NBS-LRR proteins in pathogen sensing and host defense. Nat Immunol.

[45] Schmutz J, Cannon SB, Schlueter J, Ma J, Mitros T, Nelson W (2010). Genome sequence of the palaeopolyploid soybean. Nature.

[46] Howe GA, Jander G (2008). Plant immunity to insect herbivores. Annu Rev Plant Biol.

[47] Milligan SB, Bodeau J, Yaghoobi J, Kaloshian I, Zabel P, Williamson VM (1998). The root knot nematode resistance gene *Mi* from tomato is a member of the leucine zipper, nucleotide binding, leucine-rich repeat family of plant genes. Plant Cell.

[48] Brotman Y, Silberstein L, Kovalski I, Perin C, Dogimont C, Pitrat M (2002). Resistance gene homologues in melon are linked to genetic loci conferring disease and pest resistance. Theor Appl Genet.

[49] Klingler J, Creasy R, Gao LL, Nair RM, Calix AS, Jacob HS (2005). Aphid resistance in *Medicago truncatula* involves antixenosis and phloem-specific, inducible antibiosis, and maps to a single locus flanked by NBS-LRR resistance gene analogs. Plant Physiol.

[50] Kim KS, Hill CB, Hartman GL, Hyten DL, Hudson ME, Diers BW (2010). Fine mapping of the soybean aphid-resistance gene *Rag*2 in soybean PI 200538. Theor Appl Genet.

[51] Kim KS, Hill CB, Hartman GL, Mian MAR, Diers BW (2008). Discovery of soybean aphid biotypes. Crop Sci.

[52] Andrews S. FastQC: A quality control tool for high throughput sequence data. 2010. Available at: https://www.bioinformatics.babraham.ac.uk/projects/fastqc/. Accessed 15 June 2017.

[53] Martin M (2011). Cutadapt removes adapter sequences from high-throughput sequencing reads. EMBnet J.

[54] Kim D, Pertea G, Trapnell C, Pimentel H, Kelley R, Salzberg SL (2013). TopHat2: accurate alignment of transcriptomes in the presence of insertions, deletions and gene fusions. Genome Biol.

[55] Anders S, Pyl PT, Huber W (2015). HTSeq—a python framework to work with high-throughput sequencing data. Bioinformatics.

[56] Gentleman RC, Carey VJ, Bates DM, Bolstad B, Dettling M, Dudoit S (2004). Bioconductor: open software development for computational biology and bioinformatics. Genome Biol.

[57] Du Z, Zhou X, Ling Y, Zhang Z, Su Z (2010). agriGO: a GO analysis toolkit for the agricultural community. Nucleic Acids Res.

[58] Rozen S, Skaletsky H (2000). Primer3 on the WWW for general users and for biologist programmers. Methods Mol Biol.

[59] Schmittgen TD, Livak KJ (2008). Analyzing real-time PCR data by the comparative C_T_ method. Nat Protoc.

