# Supplementary figures and images for: Transcriptomic dynamics in soybean near-isogenic lines differing in alleles for an aphid resistance gene, following infestation by soybean aphid biotype 2
Source: BMC Genomics. 2017 Jun 23;18:472. doi: 10.1186/s12864-017-3829-9 (PMC5481885; doi:10.1186/s12864-017-3829-9)

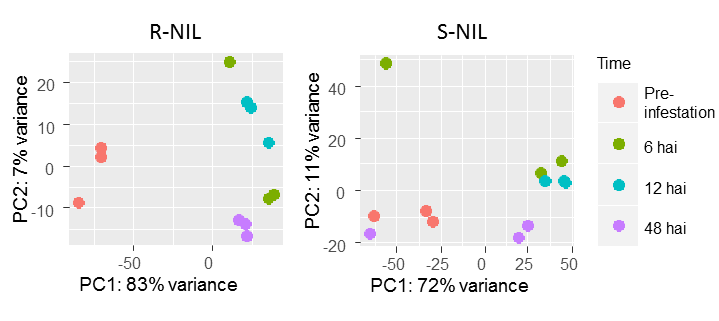

Supplement: Supplementary file 1 — Principle component analysis of 24 cDNA library samples. (TIFF 46 kb) [file 12864_2017_3829_MOESM1_ESM.tif]
